# Deep Brain Stimulation Before Anterior Cervical Discectomy and Fusion for a Patient With Cervical Dystonia and Cervical Myelopathy: A Case Report

**DOI:** 10.7759/cureus.46221

**Published:** 2023-09-29

**Authors:** Michael Folse, Ryan Diaz, Racheal Peterson, Jamie Toms

**Affiliations:** 1 Department of Neurosurgery, Louisiana State University Health Sciences Center, Shreveport, USA

**Keywords:** anterior cervical discectomy and fusion, cervical myelopathy, cervical dystonia, globus pallidus internus, deep brain stimulation

## Abstract

Cervical dystonia with concurrent cervical myelopathy is a challenging pathology that requires thoughtful management. A 46-year-old female was referred to our center with this presentation. We elected to perform bilateral globus pallidus internus deep brain stimulation (DBS-GPi) prior to C5 to C7 anterior cervical discectomy and fusion (ACDF) to avoid the potential for dystonic movements to negatively impact cervical fusion. The patient was followed up at three months post C5 to C7 ACDF and nine months post DBS-GPi with complete control of tremor and no radiographic evidence of hardware loosening or malalignment. Though this strategy was successful in treating both our patient’s cervical myelopathy and cervical dystonia, larger studies need to be conducted to optimize the treatment of patients presenting with these concurrent pathologies.

## Introduction

Dystonic movement disorders such as cervical dystonia may present with cervical myelopathy. Epidemiologic evidence has revealed that degenerative disorders of the spine associated with dystonia occur earlier in life than normal physiological aging, and disease is often seen at higher cervical levels [1]. This presentation can create challenges in the surgical management of myelopathy due to the effects of repetitive involuntary contraction of the cervical musculature on the spinal fusion process. Therefore, management of the pathophysiology in this subset of patients requires unique considerations. Several studies have reported the use of halo vests and botulinum toxin to decrease neck movement in patients following cervical fusion with mixed results [2-5]. For severe refractory cases of cervical dystonia, muscle division and intrathecal baclofen pumps are potential surgical treatments [6]. Deep brain stimulation (DBS) of bilateral globus pallidus internus (GPi) nuclei is an option that may offer a long-term solution. A recent Cochrane review found that DBS-GPi for dystonia may reduce both the severity of symptoms and improve functionality, although more research is needed to evaluate its long-term success and to determine how it may best be applied to different populations by age [7]. This case report represents an important treatment paradigm in patients with concurrent cervical dystonia and cervical myelopathy.

## Case presentation

**Figure 1:**
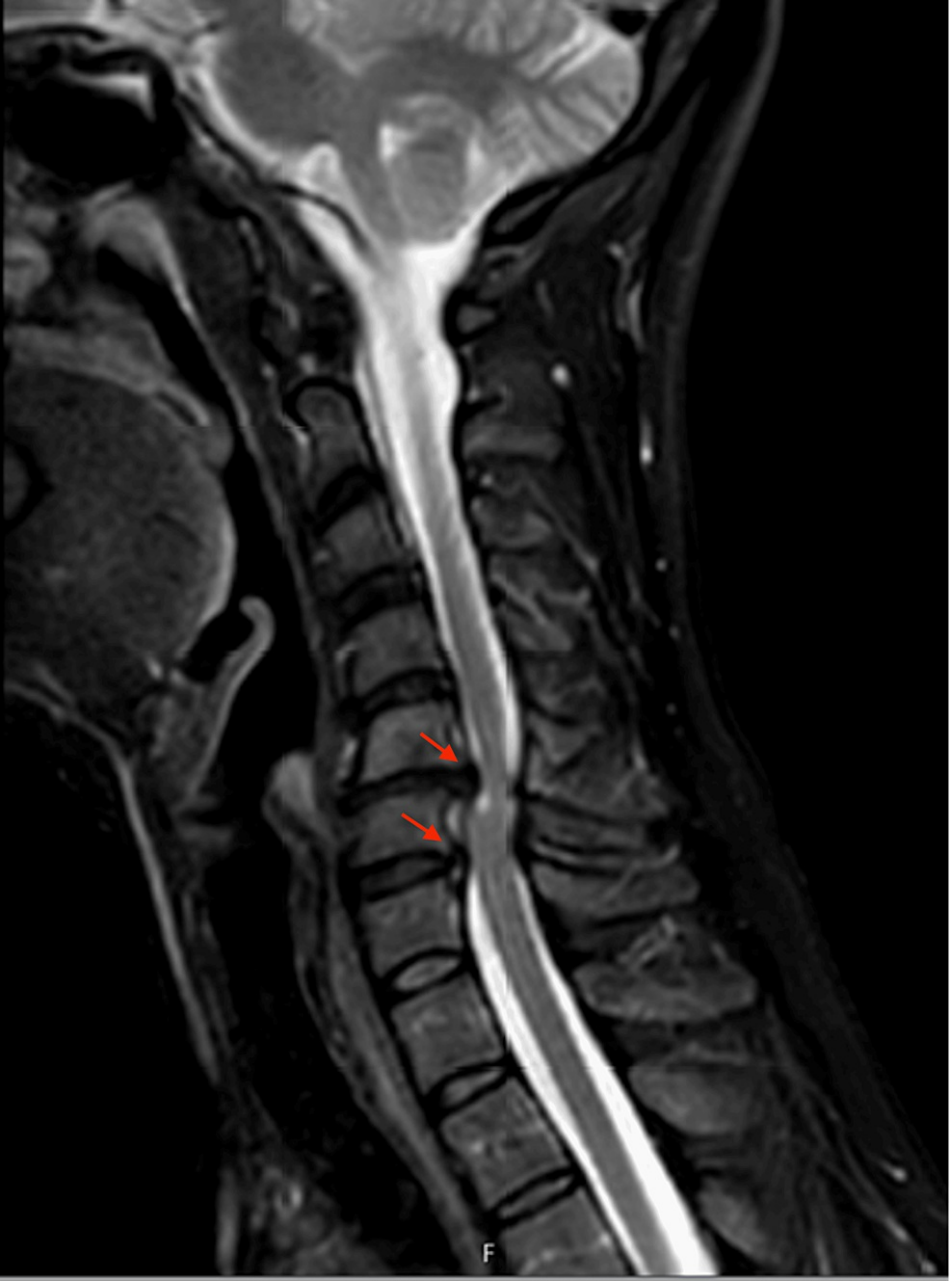
Sagittal T2 cervical MRI shows cervical myelopathy at C5-C6 and C6-C7 levels

**Figure 2:**
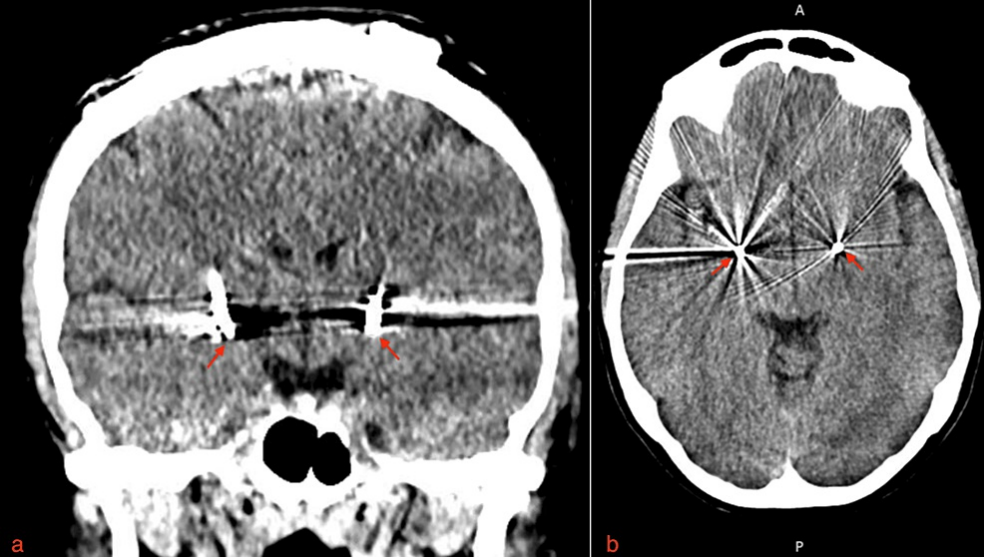
Coronal view (a) and axial view (b) of head CT depict bilateral deep brain stimulation electrodes placed in the globus pallidus internus

**Figure 3:**
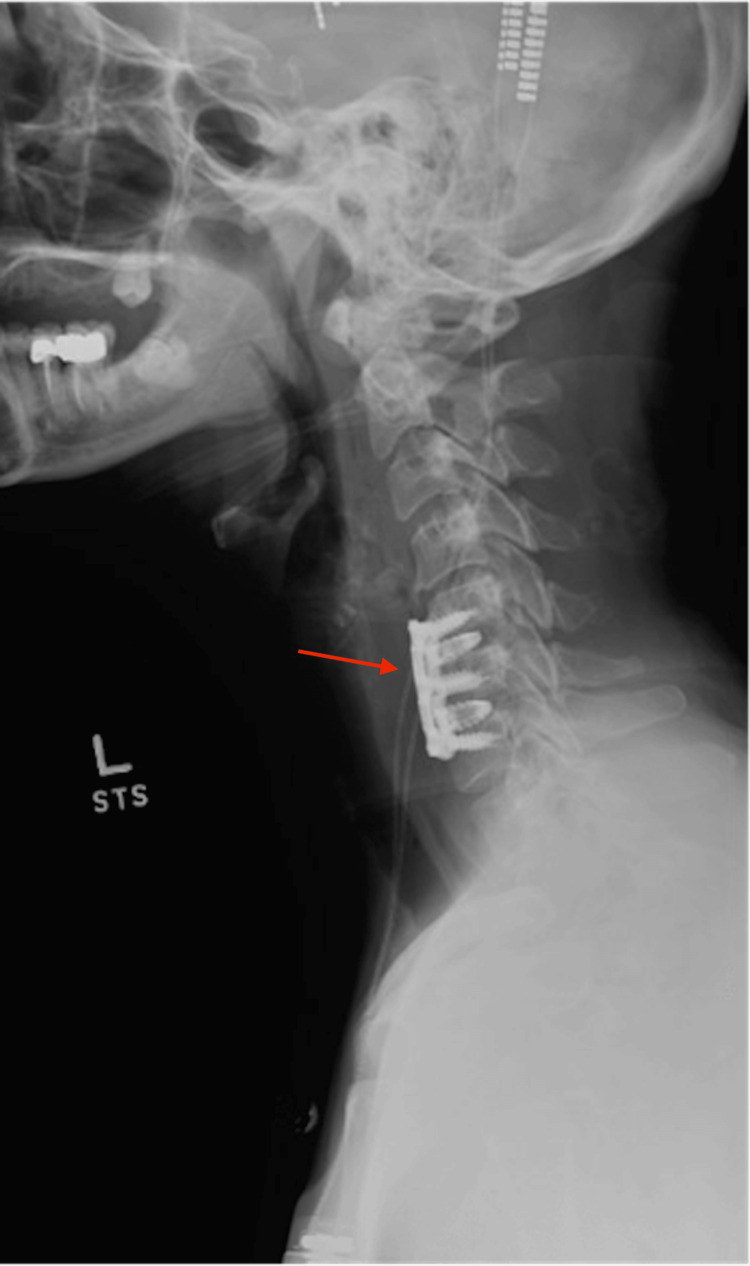
X-ray of the neck shows the placement of anterior cervical discectomy and fusion hardware with no loosening or malalignment

A 46-year-old female with a primary complaint of neck pain, bilateral upper extremity radiculopathy, paresthesia, and myelopathic gait was referred to our center. She had also been diagnosed with cervical dystonia 15 years prior. She had trialed various medications for her dystonia over many years with no relief. She also received botulinum toxin injections, which resulted in the worsening of her symptoms. The patient also had significant cervical myelopathy due to disc herniations at C5-C6 and C6-C7 with myelomalacia on cervical MRI seen in Figure 1. She was deemed an appropriate candidate for C5-C6 and C6-C7 anterior cervical discectomy and fusion (ACDF); however, due to the presence of severe dystonia, there was a high risk of pseudoarthrosis and hardware failure after cervical fusion. Thus, the patient was advised to proceed with treatment of the dystonia prior to treatment of the cervical myelopathy to decrease motion and improve her chance of fusion. She underwent bilateral DBS-GPi surgery, shown in Figure 2. The patient was brought back for stage 2 battery placement, which provided significant relief of her dystonia once turned on. At three months follow-up post DBS, the patient reported a 75% reduction in tremors but noted persistent tingling in her fingers. At six months post DBS-GPi, she underwent ACDF at C5-C6 and C6-C7. At three months follow-up post ACDF, the patient reported significant improvement in her pain, paresthesia, and myelopathic symptoms. Radiographic examination showed no evidence of hardware loosening or malalignment, which can be appreciated in Figure 3. She does not have any tremors and is content with her current DBS settings.

## Discussion

There is a small but growing body of evidence that suggests the use of DBS and surgical fixation of the spine may provide long-lasting symptom resolution for carefully selected patients. Bilateral DBS-GPi has been shown to improve symptoms of cervical dystonia for over a decade after implantation [8]. It may also be important to intervene early in patients identified as having cervical dystonia as a cause of cervical myelopathy. Chen and Wang et al. reported a series of 10 patients with cervical dystonia and cervical myelopathy who underwent spine fusion surgery without DBS, and patients who already had moderate-severe disability before spine surgery did not show improvement in function [9]. Tonomura et al. have described the use of DBS to help patients with cervical spine disease such as atlantoaxial rotary subluxation presenting with cervical dystonia [10]. However, our case is unique in that the cervical disease was much lower at the C5-C7 levels, which is less commonly seen in cervical dystonia [1]. There have only been small studies, such as case series and small prospective trials, in the literature that have reported cervical myelopathy in patients with dystonia underlining the importance of larger investigations to assess the optimal treatment strategy for these patients [2]. Krauss et al. conducted a prospective study of patients with cervical dyskinesia, which included three patients who also had cervical myelopathy, who underwent DBS-GPi in addition to spine surgery and found significant improvement in their dyskinesias and quality of life while halting the progression of their myelopathy [11].

## Conclusions

The co-occurrence of both a dystonic movement disorder and cervical myelopathy presents a unique challenge in terms of surgical management. This case presents a viable surgical strategy for managing this challenging pathology in patients whose symptoms are refractory to medical management for cervical dystonia with concurrent myelopathy. Larger clinical trials could help further establish this paradigm as an effective treatment for these patients while also elucidating the finer details of managing specific deformities in the cervical spine in this concurrent pathology.
